# Prediction of Chronic Periodontitis Severity Using Machine Learning Models Based On Salivary Bacterial Copy Number

**DOI:** 10.3389/fcimb.2020.571515

**Published:** 2020-11-16

**Authors:** Eun-Hye Kim, Seunghoon Kim, Hyun-Joo Kim, Hyoung-oh Jeong, Jaewoong Lee, Jinho Jang, Ji-Young Joo, Yerang Shin, Jihoon Kang, Ae Kyung Park, Ju-Youn Lee, Semin Lee

**Affiliations:** ^1^ Department of R&D, Helixco Inc., Ulsan, South Korea; ^2^ College of Pharmacy and Research Institute of Life and Pharmaceutical Sciences, Sunchon National University, Suncheon, South Korea; ^3^ Department of Biomedical Engineering, College of Information-Bio Convergence Engineering, Ulsan National Institute of Science and Technology (UNIST), Ulsan, South Korea; ^4^ Korean Genomics Center, UNIST, Ulsan, South Korea; ^5^ Department of Periodontology, Dental and Life Science Institute, Pusan National University, School of Dentistry, Yangsan, South Korea; ^6^ Department of Periodontology and Dental Research Institute, Pusan National University Dental Hospital, Yangsan, South Korea

**Keywords:** chronic periodontitis, multiplex qPCR, machine learning, severity prediction, salivary bacterial copy number, slight periodontitis

## Abstract

Periodontitis is a widespread chronic inflammatory disease caused by interactions between periodontal bacteria and homeostasis in the host. We aimed to investigate the performance and reliability of machine learning models in predicting the severity of chronic periodontitis. Mouthwash samples from 692 subjects (144 healthy controls and 548 generalized chronic periodontitis patients) were collected, the genomic DNA was isolated, and the copy numbers of nine pathogens were measured using multiplex qPCR. The nine pathogens are as follows: *Porphyromonas gingivalis* (Pg), *Tannerella forsythia* (Tf), *Treponema denticola* (Td), *Prevotella intermedia* (Pi), *Fusobacterium nucleatum* (Fn), *Campylobacter rectus* (Cr), *Aggregatibacter actinomycetemcomitans* (Aa), *Peptostreptococcus anaerobius* (Pa), and *Eikenella corrodens* (Ec). By adding the species one by one in order of high accuracy to find the optimal combination of input features, we developed an algorithm that predicts the severity of periodontitis using four machine learning techniques. The accuracy was the highest when the models classified “healthy” and “moderate or severe” periodontitis (H vs. M-S, average accuracy of four models: 0.93, AUC = 0.96, sensitivity of 0.96, specificity of 0.81, and diagnostic odds ratio = 112.75). One or two red complex pathogens were used in three models to distinguish slight chronic periodontitis patients from healthy controls (average accuracy of 0.78, AUC = 0.82, sensitivity of 0.71, and specificity of 0.84, diagnostic odds ratio = 12.85). Although the overall accuracy was slightly reduced, the models showed reliability in predicting the severity of chronic periodontitis from 45 newly obtained samples. Our results suggest that a well-designed combination of salivary bacteria can be used as a biomarker for classifying between a periodontally healthy group and a chronic periodontitis group.

## Introduction

Periodontitis is a multifactorial and multibacterial disease that occurs in the dental supporting tissues, involving a complex relationship between oral microorganisms organized in the subgingival biofilm and the homeostatic processes in the host. It causes inflammatory changes in the tooth supporting tissue, which is made up of the gingiva, periodontal ligament, cementum, and alveolar bone, and leads to tooth loss when not controlled (Pihlstrom et al., 2005). Moreover, periodontitis has been shown to be associated with several other severe health issues, such as coronary artery disease (Humphrey et al., 2008), diabetes (Preshaw et al., 2012), premature birth (Walia and Saini, 2015), and rheumatoid arthritis (Araújo et al., 2015). These associations have led to a change in the perception that the oral cavity is organically linked to the systemic physiology rather than just to an isolated organ (Sabharwal et al., 2018). Therefore, in order to control periodontitis associated with oral and systemic health, therapeutic intervention should be performed at an early stage. In addition, early detection and diagnosis of the disease is important because it may guarantee less-invasive, less-costly treatment than that of conventional dental care.

Recently, a new method that uses saliva for the diagnosis of periodontitis has attracted a lot of attention (Korte and Kinney, 2016). While traditional diagnostic methods, which use periodontal probes, measure the extent of tissue destruction due to disease progression, diagnostic methods that use saliva can detect real-time changes in the periodontium. In addition, the salivary diagnostic method is simple to perform, meaning the procedure can be carried out by any dental staff. Ultimately, it has the advantage of being non-invasive and less painful for the patient in contrast to traditional clinical diagnoses that measure pocket depth (Fuentes et al., 2014). Several studies have reported that not only the presence of bacteria in saliva but also the number of salivary bacteria is related to periodontitis and clinical variables of periodontitis (Könönen et al., 2007; Paju et al., 2009; Lundmark et al., 2019). Morozumi et al. evaluated the amount of bacteria in saliva and IgG titers in serum to monitor the progression of chronic periodontitis (Morozumi et al., 2016). Paque et al. recently reported that the strongest differences in eight bacterial targets (*C. rectus*, *T. forsythia*, *P. gingivalis*, *S. mutans*, *F. nucleatum*, *T. denticola*, *P. intermedia*, and oral *Lactobacilli*) were found between healthy controls and periodontitis patients by their newly developed qPCR analysis of saliva (Paque et al., 2020).

A symbiotic relationship between normal oral flora and host is essential for homeostasis in the oral cavity. Periodontitis initiates following the destruction of the host-microbe homeostasis, which is caused by the dysbiosis of microbiota (Kilian et al., 2016). Recent research on the relationship between microbiota and periodontal health or periodontitis has mainly focused on the 40 or so previously studied representative bacterial species that are most prominent in the periodontitis patients (Colombo et al., 2002; Teles et al., 2013). The five major complexes reported by Socransky et al. (Socransky et al., 1998) have been investigated to understand the nature of the microbial complexes in subgingival biofilm. These studies (Colombo et al., 2002; Teles et al., 2013) reported that three species from the red complex (*P*. *gingivalis*, *T*. *denticola*, and *T*. *forsythia*) could be defined as the main periodontal pathogens. Some from the orange complex, such as *Prevotella intermedia*, *Parvimonas micra*, *Fusobacterium nucleatum*, and *Eubacterium nodatum* and the *Aggregatibacter actinomycetemcomitans* from the “other” complex, have been associated with periodontal disease in combination with the three species of the red complex (Teles et al., 2013). In addition, these 40 representative bacterial species are known to occupy 55–60% of bacteria in subgingival plaque (Socransky and Haffajee, 2005).

In a study analyzing salivary pathogen burden in periodontitis, combining salivary *P. gingivalis* and *T. forsythia* was the most accurate biomarker for the diagnosis of periodontitis amongst the various combinations of four periodontal pathogens (*P*. *gingivalis*, *T*. *forsythia*, *P*. *intermedia*, and *A*. *actinomycetemcomitans*) (Salminen et al., 2015). This suggests that the cumulative strategy appears to be useful in the analysis of salivary bacteria as biomarkers of periodontitis.

However, most studies that have reported on the relationships between combinations of periodontal pathogens and periodontitis have limitations in their study designs, in that the periodontal pathogen combinations were made by randomized grouping into two or three species (Ready et al., 2008; Paju et al., 2009; Salminen et al., 2015). Nowadays, machine learning is drawing attention as a data analysis method that can be used to find out patterns in various values and to make risk predictions for several diseases, such as cancer (Kourou et al., 2015). Also, machine learning can develop algorithms by learning through the tagged examples instead of performing clear, predefined routines (Noble, 2006; Chan et al., 2016). This technique is useful in the biomedical field, as it can be used to discover the relative biomarkers for diagnosis and prediction of disease. A widely used machine learning technique in the biomedical field is the support vector machine (SVM) (Noble, 2006). It has been used for the gene-expression profiling of samples derived from tumors and for tumor marker detection for different types of cancers (Li et al., 2012; Huang et al., 2018). This method is becoming popular in dentistry too, with a handful of studies using significant classifiers, such as SVMs, artificial neural networks (ANNs), and random forest, to identify the relationship between periodontitis and bacteria (Szafranski et al., 2015; Chen et al., 2018).

Prior to applying machine learning techniques in this study, we quantified the salivary bacterial copy number from healthy controls and chronic periodontitis patients using a culture-independent molecular method based on PCR. Next, we determined the core bacteria most relevant to periodontitis according to the severity of disease and then used machine learning techniques to add bacteria sequentially in a way that maximizes accuracy to find the best combinations. In this process, neural network, SVM, regularized logistic regression, and random forest were applied to find the optimal model. The hypothesis tested was that bacterial combinations made by machine learning could be a biomarker with the ability to differentiate between healthy controls and patients with differing severities of periodontitis.

## Materials and Methods

### Study Subjects and Clinical Examination

This study included 692 subjects (144 periodontally healthy controls and 548 generalized chronic periodontitis patients) who visited the Department of Periodontics at Pusan National University Dental Hospital, between August 2016 and March 2019. The study protocol was approved by the Institutional Review Board of Pusan National University Dental Hospital (PNUDH-2016-019). All subjects received complete information regarding the objectives and procedures of this study and provided written informed consent.

The diagnosis of healthy controls and patients with generalized chronic periodontitis was based on clinical examination and X-ray viewing (panoramic view or standard view), and the criteria followed the classification of the American Periodontal Society Workshop in 1999 (Armitage, 1999). The severity of chronic periodontitis was categorized on the basis of clinical attachment loss as follows: slight = 1 or 2 mm, moderate = 3 or 4 mm, and severe ≥ 5 mm.

The following patients were excluded: 1) those who received periodontal treatment within the past 6 months; 2) women who were pregnant or breastfeeding; 3) those who refused to sign the informed consent form.

The clinical attachment level (CAL), probing depth (PD), gingival index (GI), and plaque index (PI) were measured during the clinical evaluation. The CAL and PD were measured using a periodontal probe (PGF-W, Osung, Kwangmyung, South Korea). The CAL and PD were determined by the distance from reference point to bottom of pocket using reference as cemento-enamel junction and gingival margin respectively (Pihlstrom et al., 1984). The activity of periodontal tissue inflammation was evaluated by GI (Loe, 1967). The PI is an indicator of oral hygiene and is determined by the O’Leary plaque index (O’Leary et al., 1972). All measurements were performed by two experienced periodontists.

The dental and smoking statuses of participants were gathered from questionnaires, including information such as oral care (use of dental floss or mouthwash) and gargling frequency per day. Smoking status was categorized into three classes to distinguish present smokers, former smokers (those who had quit more than six months previously) smokers, and those who had never smoked.

### Analysis of Bacterial Copy Number Using Multiplex qPCR

Collection of mouthwash samples and DNA extraction were performed as in the Materials and Methods section of the previous study (Kim et al., 2018). The multiplex qPCR system was optimized for nine pathogens after the construction of standard curves for each pathogen. The nine pathogens were as follows: *Porphyromonas gingivalis* (*Pg*), *Tannerella forsythia* (*Tf*), *Treponema denticola* (*Td*), *Prevotella intermedia* (*Pi*), *Fusobacterium nucleatum* (*Fn*), *Campylobacter rectus* (*Cr*), *Aggregatibacter actinomycetemcomitans* (*Aa*), *Peptostreptococcus anaerobius* (*Pa*), and *Eikenella corrodens* (*Ec*). The aim of the previous study was to develop a grading system by analyzing the copy numbers of multiple pathogens in 170 mouthwash samples (64 healthy controls and 106 chronic periodontitis patients). The bacterial copy numbers in these samples and those in the 567 newly obtained samples were both used in this study.

### Classifier Model Construction

To predict the severity of periodontitis using the bacterial copy number, we performed three binary classification tasks (healthy controls (H) vs. slight chronic periodontitis patients (Sli), healthy controls (H) vs. moderate or severe chronic periodontitis patients (M-S), healthy controls (H) vs. all chronic periodontitis patients (Sli-M-S)). We utilized four well-known machine learning algorithms: neural network, random forest, support vector machines with linear kernel, and regularized logistic regression in R caret package (version 6.0-84) (Kuhn, 2008). Among the various neural network analysis methods, we used feed-forward neural networks with a single hidden layer implemented in the caret package. For the classification analysis, the concentration of the bacteria was log-transformed. We created a pseudo-count by adding 1 to the real copy number before doing the log transformation. The patient dataset was randomly divided into five equal-sized subsamples (5-fold cross validation). Four subsets were trained to make a prediction model, and one subset was used as the validation set for testing the model. This process was repeated five times, using a different subsample each time as the validation set. The average accuracy of the five tests was used as the accuracy of the model while using balanced accuracy as a way to compensate for imbalanced test sets (Brodersen et al., 2010).

### Finding the Best Pathogen Combination

The first step was to predict the accuracy of each pathogen in the classification process using the four machine learning models. Next, we chose the pathogen with the highest accuracy, added the other pathogens to it one by one, predicted the accuracy of the two pathogens, and selected the combination showing the highest accuracy at each step. By repeating this method, we found the combination of pathogens with the highest accuracy and the corresponding parameters for each machine learning model.

### Calculating the Diagnostic Odds Ratio

As an indicator of model performance, we used the diagnostic odds ratio (DOR) (Glas et al., 2003). The DOR is defined as the ratio of the odds of the test being positive in patients relative to the odds of the test being positivity in healthy people. We calculated DOR with the following formula:$\text{DOR}=\frac{\text{sensitivity}\times \;\text{specificity}}{\text{(1}-\text{sensitivity)}\times (1-\text{specificity)}}$

In general, a higher DOR indicates better model performance. If the DOR is 1, it means the test cannot differentiate between the diseased group and the healthy group.

### Validation of Model Accuracy

We obtained 45 additional mouthwash samples from Pusan National University Dental Hospital. The accuracy of the model was validated using these 45 samples with the optimal combination of pathogens and optimal machine learning parameter values.

### Sample Visualization Using T-Distributed Stochastic Neighbor Embedding (t-SNE)

T-SNE is a technique to reduce the dimensions of a high-dimensional dataset into low-dimensions (usually two or three dimensions) for visualization (Maaten and Hinton, 2008). We drew a t-SNE plot using the four python packages: Matplotlib (Hunter, 2007), Pandas (McKinney, 2010), Scipy (Virtanen et al., 2019), and Scikit-learn (Pedregosa et al., 2011). The Log_10_ (copy number) information of nine pathogens was used as input data. To perform t-SNE analysis, the copy numbers of the nine pathogens ​​were standardized.

### Statistical Analysis

The reproducibility of two separate investigator and intra-investigator assessments were evaluated using Cohen’s kappa index. The intra- and inter-examiner agreements were 0.81 and 0.72, respectively. All statistical analyses were performed using SigmaPlot 13.0 software (Systat Software Inc., San Jose, USA) or R statistical software (https://www.R-project.org/). The differences in the characteristics and detection rates of the bacteria between the healthy controls and periodontitis patients were analyzed using the chi-squared or Fisher’s exact tests. Fisher’s exact tests were performed when the criteria for the chi-squared test were not fulfilled. Comparisons among all groups were performed using the Kruskal-Wallis test, and Dunn’s test was used to correct for multiple comparisons. P-values were considered statistically significant when the p-value was less than 0.05, and marginally significant when P > 0.05, up to 0.1.

## Results

### Characteristics of the Study Subjects

**Figure 1:**
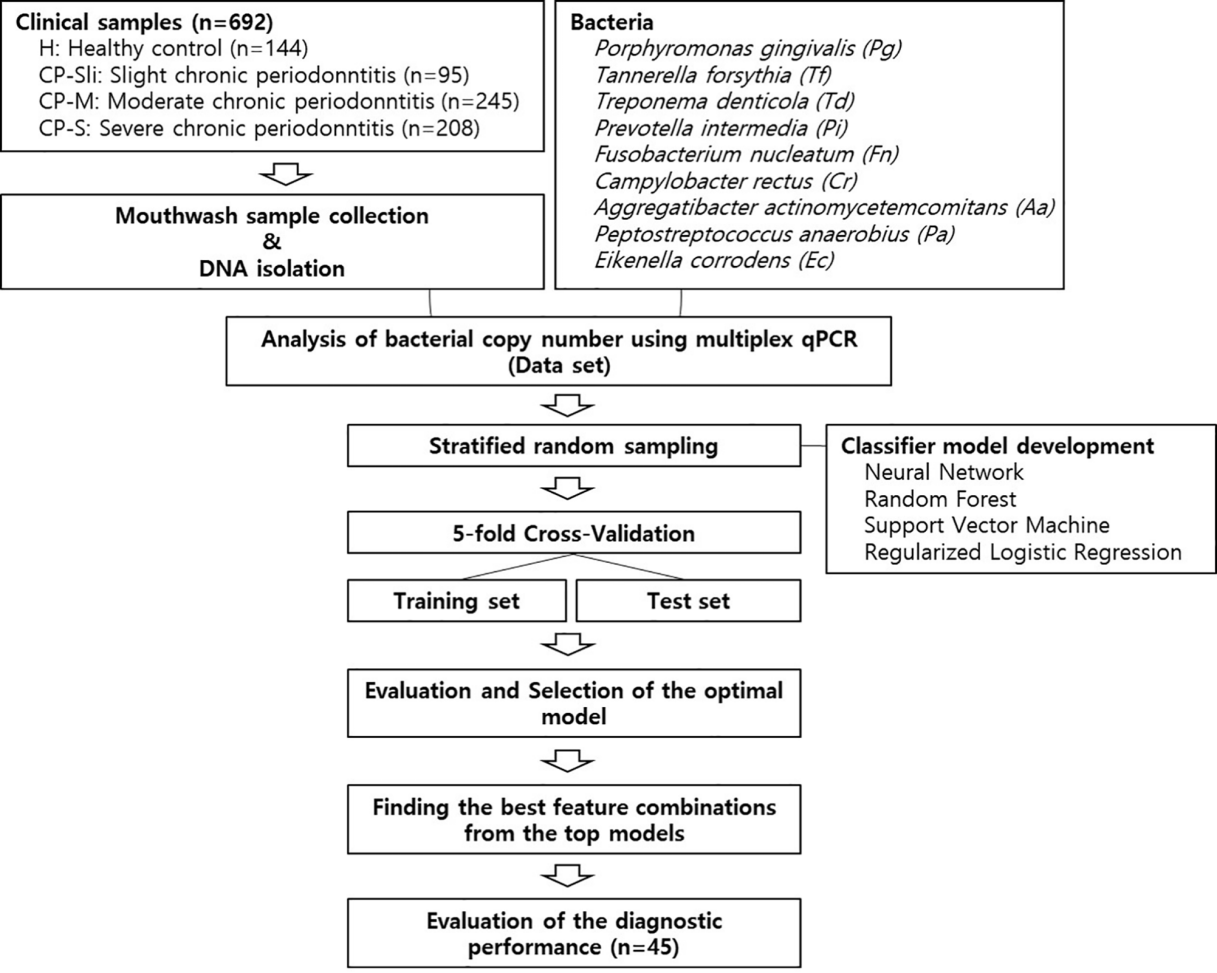
Machine learning workflow for predicting the severity of chronic periodontitis using qPCR data.

**Figure 1** shows the overall workflow for predicting the severity of chronic periodontitis based on salivary bacterial copy number. Mouthwash samples were collected from 144 periodontally healthy subjects and 548 generalized chronic periodontitis patients. Of the 548 patients, 95 were diagnosed as slight (CP-Sli), 245 as moderate (CP-M), and 208 as severe chronic periodontitis (CP-S). The characteristics of the study population are shown in **Table 1** including demographic, and clinical information, along with oral hygiene behaviors. The patients with periodontitis were considerably older and had higher levels of periodontal clinical parameters with statistical significance when compared to the controls (all P-values < 0.001). Smoking history and oral hygiene behaviors of the periodontitis patients were also significantly different from those of the healthy controls (all P-values < 0.001).

**Table 1:** Characteristics of healthy subjects and periodontitis patients (n = 692).

Characteristics	Healthy (n = 144)	Chronic periodontitis			P-value	
		Slight(n = 95)	Moderate(n = 245)	Severe(n = 208)		
**Sex**						
Male	70 (48.6%)	42 (44.2%)	126 (51.4%)	123 (59.1%)	0.065^a^	
Female	74 (51.4%)	53 (55.8%)	119 (48.6%)	85 (40.9%)		
**Age (yr)**						
20-29	79 (54.9%)	6 (6.3%)	3 (1.2%)	0 (0.0%)	<0.001^a^	
30-39	42 (29.2%)	20 (21.1%)	18 (7.3%)	11 (5.3%)		
40-49	3 (2.1%)	20 (21.1%)	45 (18.4%)	48 (23.1%)		
50-59	10 (6.9%)	26 (27.4%)	85 (34.7%)	97 (46.6%)		
≥60	10 (6.9%)	23 (24.2%)	94 (38.4%)	52 (25.0%)		
**Clinical attachment level (mm)**						
Mean ± SD	2.46 ± 0.29	2.82 ± 0.40	3.49 ± 0.67	4.47 ± 1.18	<0.001^b^	
Median (IQR)	2.47 (2.24-2.66)	2.80 (2.53-3.03)	3.40 (3.05-3.79)	4.26 (3.71-5.09)		
**Pocket depth (mm)**						
Mean ± SD	2.43 ± 0.29	2.67 ± 0.36	3.16 ± 0.60	3.90 ± 0.89	<0.001^b^	
Median (IQR)	2.44 (2.23-2.64)	2.68 (2.44-2.88)	3.13 (2.82-3.47)	3.85 (3.29-4.32)		
**Plaque index**						
Mean ± SD	17.00 ± 14.68	32.36 ± 21.33	46.12 ± 25.47	52.78 ± 25.76	<0.001^b^	
Median (IQR)	13.84 (7.14-22.24)	30.95 (15.83-48.28)	46.88 (24.50-61.61)	50.00 (31.96-71.76)		
**Gingival index**						
Mean ± SD	0.08 ± 0.16	0.39 ± 0.40	0.70 ± 0.49	1.01 ± 0.55	<0.001^b^	
Median (IQR)	0.03 (0.00-0.10)	0.27 (0.14-0.51)	0.63 (0.29-1.05)	0.99 (0.50-1.50)		
**Smoking history**						
Never	120 (83.3%)	69 (72.6%)	144 (58.8%)	97 (46.6%)	<0.001^a^	
Former	19 (13.2%)	17 (17.9%)	52 (21.2%)	51 (24.5%)		
Daily	5 (3.5%)	9 (9.5%)	49 (20.0%)	60 (28.8%)		
**Additional oral care (dental floss, mouthwash)**						
Yes	113 (78.5%)	69 (72.6%)	147 (60.0%)	113 (54.3%)	<0.001^a^	
No	31 (21.5%)	26 (27.4%)	98 (40.0%)	95 (45.7%)		
**Toothbrushing frequency per day**						
≥3 times	114 (79.2%)	53 (55.8%)	116 (47.3%)	95 (45.7%)	<0.001^a^	
≤2 times	30 (20.8%)	42 (44.2%)	129 (52.7%)	113 (54.3%)	

^a^Chi-squared test, ^b^Kruskal-Wallis One Way Analysis of Variance on Ranks.

### Detection Rate and Copy Numbers of Nine Pathogens

**Figure 2:**
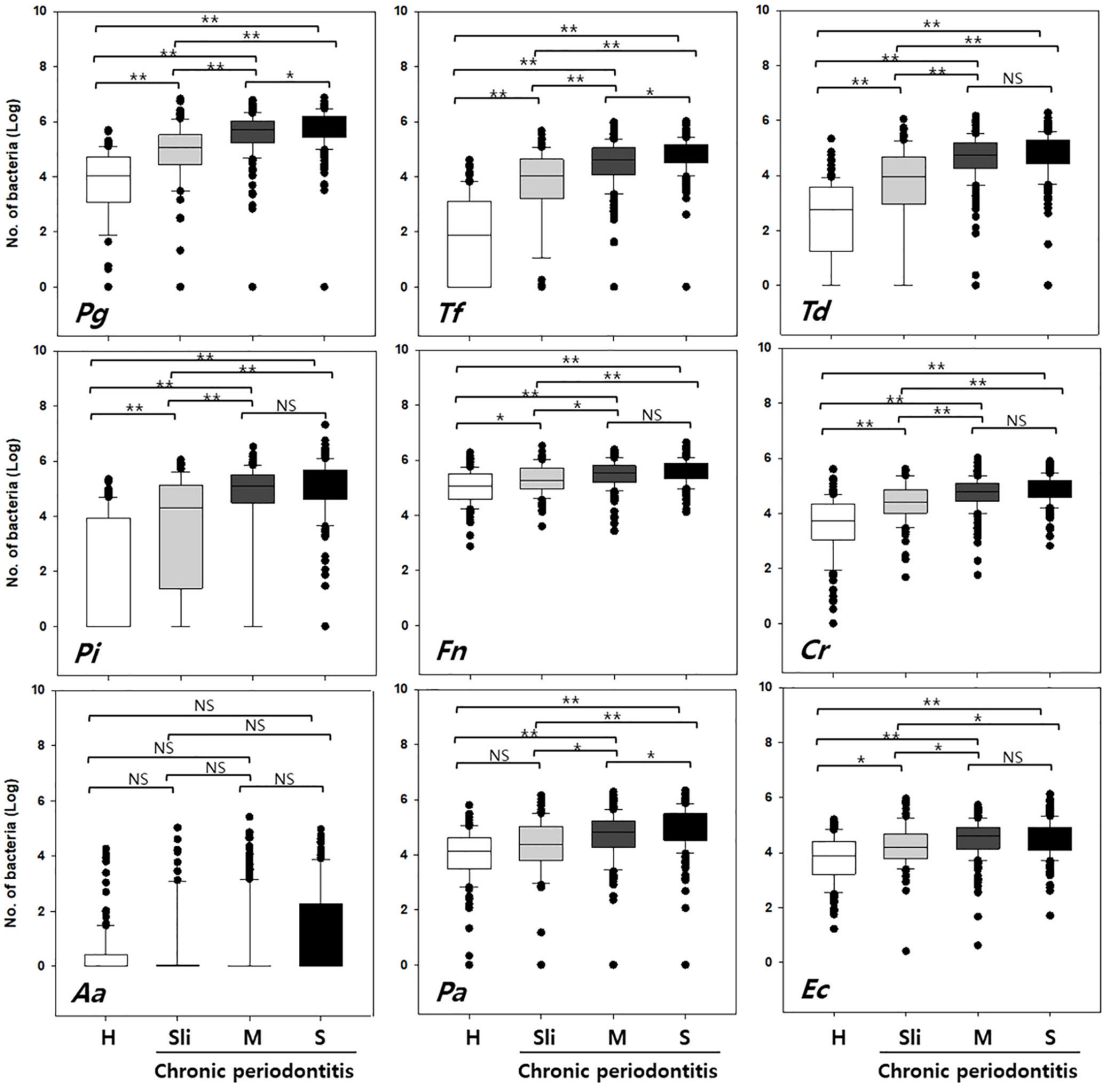
Quantification of the copy numbers of periodontal pathogens in mouthwash samples from healthy controls and chronic periodontitis patients. Whisker box plots indicate the distributions of copy numbers in each group. Pg, Porphyromonas gingivalis; Tf, Tannerella forsythia; Td, Treponema denticola; Pi, Prevotella intermedia; Fn, Fusobacterium nucleatum; Cr, Campylobacter rectus; Aa, Aggregatibacter actinomycetemcomitans; Pa, Peptostreptococcus anaerobius; Ec, Eikenella corrodens. H, Healthy; Sli, Slight; M, Moderate; S, Severe. *p < 0.05, **p < 0.001, NS, not statistically significant.

The detection rate and copy numbers of multiple pathogens were examined using multiplex qPCR in healthy controls and chronic periodontitis patients (**Table 2** and **Table S1**). The detection rates of most pathogens, except *Fusobacterium nucleatum* (*Fn*), were significantly higher in periodontitis cases in contrast to the healthy controls. *Fn* was detected in both the healthy controls and periodontitis cases, whereas Peptostreptococcus anaerobius (Pa) and Eikenella corrodens (Ec) showed slightly higher detection rates in periodontitis patients with marginal significance (P-value = 0.055 and 0.076 in chi-squared tests). Interestingly, *Tannerella forsythia* (*Tf*) was present in less than 50% of the healthy controls while it was significantly enriched in chronic periodontitis patients. Furthermore, the copy numbers of all other pathogens, except *Aggregatibacter actinomycetemcomitans* (*Aa*), were significantly higher in periodontitis patients (all P-values < 0.001 in Kruskal-Wallis test, **Figure 2**).

**Table 2:** Detection frequency and copy numbers of nine pathogens from clinical samples.

Pathogens	Detection rate; No. of subjects (%)				P-value^a^	Copy numbers; Mean ± SD, Median (IQR)				P-value^b^
	Healthy(n = 144)	Chronic periodontitis				Healthy(n = 144)	Chronic periodontitis			
		Slight(n = 95)	Moderate(n = 245)	Severe(n = 208)			Slight(n = 95)	Moderate(n = 245)	Severe(n = 208)	
***Pg***	130 (90.3%)	90 (94.7%)	242 (98.8%)	205 (98.6%)	<0.001	3.73 ± 1.43 4.05 (3.07-4.73)	4.75 ± 1.37 5.07 (4.44-5.53)	5.51 ± 0.92 5.70 (5.24-6.02)	5.70 ± 0.90 5.89 (5.44-6.18)	<0.001
***Tf***	71 (49.3%)	84 (88.4%)	239 (97.6%)	207 (99.5%)	<0.001	1.71 ± 1.58 1.89 (0.00-3.11)	3.64 ± 1.46 4.04 (3.20-4.64)	4.45 ± 0.94 4.60 (4.06-5.06)	4.78 ± 0.64 4.86 (4.50-5.18)	<0.001
***Td***	101 (70.1%)	78 (82.1%)	236 (96.3%)	204 (98.1%)	<0.001	2.45 ± 1.39 2.75 (1.24-3.58)	3.47 ± 1.72 3.94 (2.96-4.67)	4.55 ± 1.08 4.73 (4.25-5.20)	4.74 ± 0.91 4.91 (4.43-5.29)	<0.001
***Pi***	64 (44.4%)	70 (73.7%)	215 (87.8%)	202 (97.1%)	<0.001	1.81 ± 2.04 0.00 (0.00-3.95)	3.52 ± 2.15 4.32 (1.38-5.14)	4.51 ± 1.76 5.09 (4.49-5.52)	5.00 ± 1.11 5.21 (4.60-5.67)	<0.001
***Fn***	144 (100%)	95 (100%)	245 (100%)	208 (100%)	NA	5.02 ± 0.61 5.05 (4.59-5.50)	5.30 ± 0.55 5.27 (4.94-5.73)	5.47 ± 0.48 5.53 (5.19-5.80)	5.56 ± 0.45 5.55 (5.33-5.89)	<0.001
***Cr***	130 (90.3%)	94 (98.9%)	244 (99.6%)	208 (100%)	<0.001	3.52 ± 1.15 3.74 (3.06-4.33)	4.36 ± 0.74 4.42 (4.01-4.86)	4.72 ± 0.58 4.78 (4.44-5.11)	4.88 ± 0.51 4.98 (4.58-5.21)	<0.001
***Aa***	11 (7.6%)	15 (15.8%)	35 (14.3%)	52 (25.0%)	<0.001	0.45 ± 0.93 0.0056 (0.00-0.42)	0.59 ± 1.30 0.0041 (0.00-0.04)	0.55 ± 1.28 0.0025 (0.00-0.02)	0.96 ± 1.61 0.0037 (0.00-2.26)	0.107
***Pa***	137 (95.1%)	89 (93.7%)	232 (94.7%)	206 (99.0%)	0.055	3.94 ± 1.11 4.15 (3.47-4.62)	4.17 ± 1.30 4.39 (3.80-5.03)	4.53 ± 1.28 4.82 (4.28-5.24)	4.92 ± 0.87 5.05 (4.53-5.49)	<0.001
***Ec***	139 (96.5%)	94 (98.9%)	243 (99.2%)	207 (99.5%)	0.076	3.75 ± 0.85 3.88 (3.20-4.40)	4.23 ± 0.80 4.20 (3.78-4.68)	4.49 ± 0.67 4.59 (4.13-4.93)	4.51 ± 0.65 4.54 (4.08-4.92)	<0.001

^a^Chi-squared test, ^b^Kruskal-Wallis One Way Analysis of Variance on Ranks.

NA, not applicable; IQR, interquartile range.

### Machine Learning Classification With Optimal Pathogen Combinations

Four multivariate machine learning models (neural network, random forest, support vector machine, and regularized logistic regression) based on the copy numbers of nine pathogens were trained to separate the healthy controls from the patients with differing severities of periodontitis. How the optimal combination of pathogens was found is detailed in the materials and methods section.

In order to accurately distinguish between the healthy controls and chronic periodontitis patients with differing severities, the following three classification groups were used: Group 1) healthy (H) vs. moderate or severe (M-S), Group 2) H vs. all chronic periodontitis (Sli-M-S), Group 3) H vs. patients diagnosed as slight (Sli). The moderate (M) and severe (S) cases were combined because there was no significant difference in the number of copies of in six of the pathogens (*T*, *Pi*, *Fn*, *Cr*, *Aa*, and *Ec*, **Figure 2**) in those cases.

**Figure 3:**
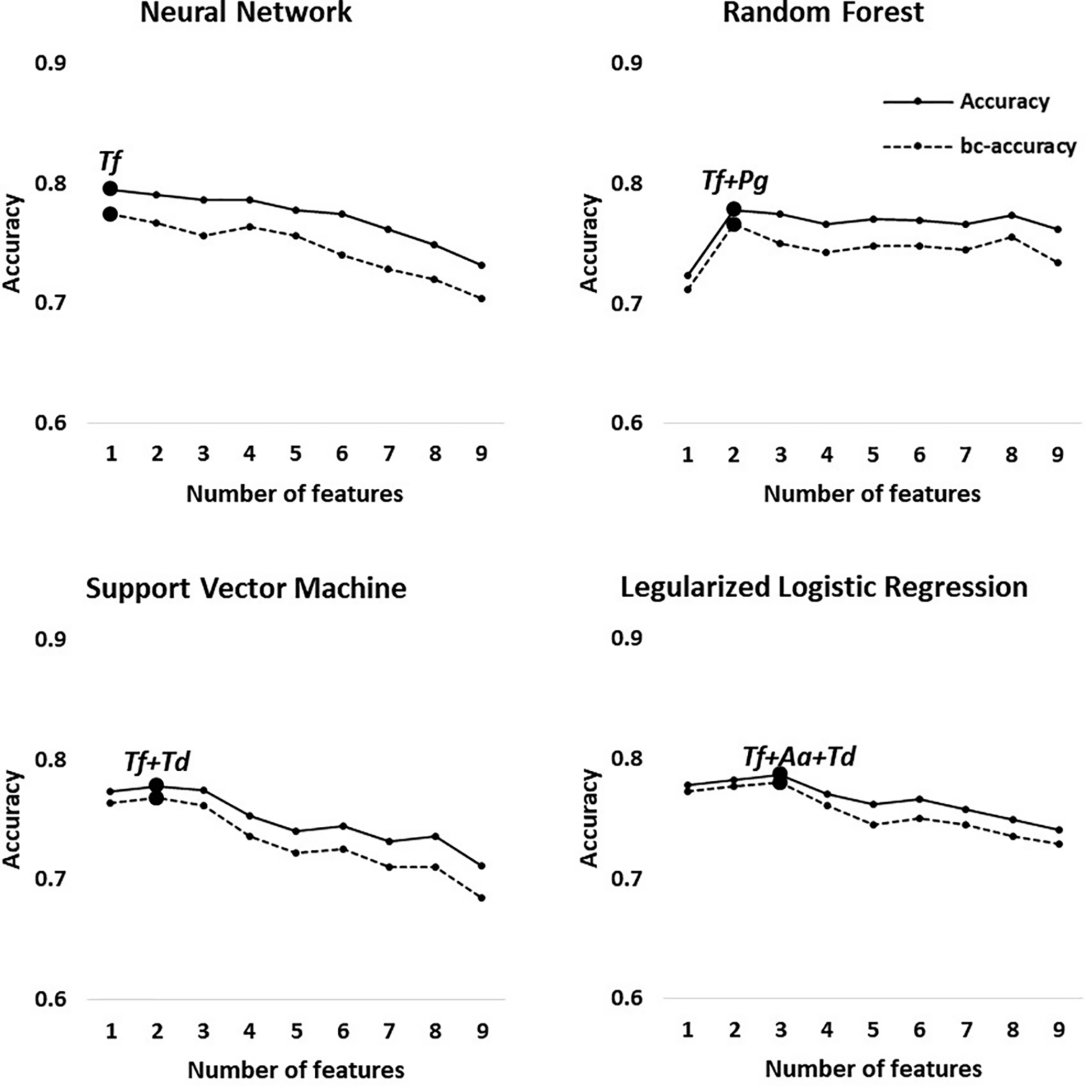
Optimal accuracy and balanced accuracy based on the number of features in Group 3 (H vs. Sli). Big circles represent the feature combination with the highest accuracy in each model.

The best feature (bacterial) combinations and their predictive accuracy and area under the curve (AUC) with multivariate models are shown in **Table 3** and **Supplementary Figure S1**. The accuracy was the highest when the models classified H and M-S (Group 1, average accuracy of four models: 0.93). The average AUC value was 0.96 with high specificity and sensitivity when distinguishing moderate or severe chronic periodontitis patients from healthy controls [odds ratio (OR): 112.75, **Supplementary Figure S1**]. In particular, *Tf* and *Pg* were included with priority in the optimal combination for all the machine learning models. The balanced accuracy, which is used to adjust unbalanced datasets, was the highest in the neural network model (0.91, **Supplementary Figure S2**). The average accuracy of the models analyzing Group 2 (H and Sli-M-S) was predicted to be 0.90 (AUC = 0.94, OR: 48.05, **Supplementary Figure S2**). The best combinations from the four prediction models all include *Tf* and *Pa*. The neural network model has the highest balanced accuracy (0.86, OR: 50.0) and the support vector machine has the lowest balanced accuracy (0.79, OR: 50.6). One or two red complex pathogens in all models that were applied to Group 3 (H and Sli), except for the regularized logistic regression model, were used to classify slight chronic periodontitis patients from healthy controls (**Table 3** and **Figure 3**).

**Table 3:** Feature combinations and their predictive accuracy with different machine learning methods.

Group		Model	Feature combination	Accuracy	Balanced accuracy	AUC (95% CI)	Sensitivity (95% CI)	Specificity(95% CI)	Odds ratio(95% CI)
1	H vs. M-S	Neural Network	*Tf+Pg+Pi+Fn+Pa+Cr+Td*	0.93	0.91	0.96(0.95–0.98)	0.95(0.92–0.98)	0.87(0.78–0.95)	127.2(67.08–241.01)
	H vs. M-S	Random Forest	*Tf+Pg+Fn+Td+Ec+Cr*	0.93	0.89	0.96(0.95–0.97)	0.96(0.92–0.99)	0.83(0.75–0.91)	117.2(61.56–223.04)
	H vs. M-S	Support Vector Machine	*Tf+Pg+Pi+Pa+Td*	0.92	0.86	0.96(0.94–0.99)	0.97(0.95–1.00)	0.74(0.65–0.83)	92(48.03–176.33)
	H vs. M-S	Regularized Logistic Regression	*Tf+Pg+Pi+Cr*	0.92	0.88	0.97(0.95–0.98)	0.97(0.95–0.99)	0.78(0.66–0.91)	114.6(59.16–222.12)
			**Average**	**0.93**	**0.88**	**0.96**	**0.96**	**0.81**	**112.75**
2	H vs. Sli-M-S	Neural Network	*Tf+Ec+Pg+Pa+Td*	0.90	0.86	0.94(0.91–0.97)	0.93(0.90–0.97)	0.79(0.68–0.89)	50.0(29.71–84.07)
	H vs. Sli-M-S	Random Forest	*Tf+Ec+Aa+Pg+Pa+Cr*	0.91	0.84	0.94(0.92–0.96)	0.95(0.91–1.00)	0.72(0.65–0.80)	48.9(28.71–83.14)
	H vs. Sli-M-S	Support Vector Machine	*Tf+Cr+Pa+Pi+Fn*	0.89	0.79	0.94(0.91–0.96)	0.97(0.93–1.00)	0.61(0.53–0.70)	50.6(27.76–92.12)
	H vs. Sli-M-S	Regularized Logistic Regression	*Tf+Cr+Pg+Pa+Aa+Fn+Pi*	0.90	0.80	0.94(0.91–0.96)	0.96(0.93–1.00)	0.64(0.59–0.69)	42.7(24.73–73.62)
			**Average**	**0.90**	**0.82**	**0.94**	**0.95**	**0.69**	**48.05**
3	H vs. Sli	Neural Network	*Tf*	0.80	0.77	0.82(0.74–0.89)	0.67(0.55–0.80)	0.88(0.82–0.93)	14.89(7.67–28.91)
	H vs. Sli	Random Forest	*Tf+Pg*	0.78	0.77	0.81(0.75–0.88)	0.71(0.57–0.84)	0.83(0.76–0.89)	12.00(6.42–22.26)
	H vs. Sli	Support Vector Machine	*Tf+Td*	0.78	0.77	0.83(0.78–0.88)	0.72(0.55–0.89)	0.82(0.77–0.86)	11.70(6.33–21.68)
	H vs. Sli	Regularized Logistic Regression	*Tf+Aa+Td*	0.79	0.78	0.82(0.77–0.87)	0.75(0.58–0.91)	0.81(0.73–0.90)	12.80(6.85–23.87)
			**Average**	**0.78**	**0.77**	**0.82**	**0.71**	**0.84**	**12.85**

AUC, area under the curve; CI, confidence interval.

In Group 1 through 3, the first high-accuracy pathogen in all groups was *Tf*, which increased the accuracy and odds ratio even further when other pathogens were added to the combination (**Figure 3** and **Supplementary Figures S2** and **S3**).

### Validation of Classification Models

To validate the performance and reliability of the results obtained from the classification models, we predicted the severity of chronic periodontitis in 45 newly obtained samples (**Table 4** and **Table S2**). Four machine learning models with the optimized parameters and feature combinations were used for validation, and the results of their performances are presented in **Table 5**. Although the overall accuracy was reduced, H and M-S was classified with 0.84 and 0.86 accuracy (sensitivity = 0.93 in both models, specificity = 0.77 and 0.82) in the neural network and random forest models, respectively. For the classification of H and Sli-M-S, the neural network model classified with 0.69 accuracy (sensitivity = 0.74, specificity = 0.64) and random forest model classified with 0.76 accuracy (sensitivity = 0.78, specificity = 0.73). H and Sli was classified with 0.63 and 0.73 accuracy (0.25 and 0.50 sensitivity, 0.77 and 0.82 specificity) in both models, respectively.

**Table 4:** Characteristics of healthy subjects and periodontitis patients (n = 45).

Characteristics	Healthy (n = 22)	Chronic periodontitis		P-value
		Slight (n = 8)	Moderate-Severe (n = 15)^C^	
**Sex**				
Male	14 (63.6%)	4 (50.0%)	6 (40.0%)	0.360^a^
Female	8 (36.4%)	4 (50.0%)	9 (60.0%)	
**Age (year)**				
20-29	2 (9.1%)	1 (12.5%)	0 (0.0%)	<0.001^a^
30-39	17 (77.3%)	2 (25.0%)	0 (0.0%)	
40-49	0 (0.0%)	4 (50.0%)	3 (20.0%)	
50-59	2 (9.1%)	0 (0.0%)	7 (46.7%)	
≥60	1 (4.5%)	1 (12.5%)	5 (33.3%)	
**Clinical attachment level (mm)**				
Mean ± SD	2.46 ± 0.19	2.92 ± 0.52^d^	3.96 ± 1.22	<0.001^b^
Median (IQR)	2.49 (2.33–2.57)	2.84 (2.43–3.31)^d^	3.72 (3.47–4.29)	
**Pocket depth (mm)**				
Mean ± SD	2.36 ± 0.19	2.52 ± 0.34d	3.36 ± 0.63	<0.001^b^
Median (IQR)	2.30 (2.22–2.49)	2.54 (2.27–2.68)^d^	3.34 (2.94–3.74)	
**Plaque index**				
Mean ± SD	25.60 ± 11.42	57.04 ± 16.98d	53.28 ± 20.07	<0.001^b^
Median (IQR)	25.89 (19.26–29.48)	57.41 (45.69–63.39)^d^	50.00 (35.71–65.52)	
**Gingival index**				
Mean ± SD	0.29 ± 0.16	0.43 ± 0.39d	0.86 ± 0.53	0.003^b^
Median (IQR)	0.30 (0.18–0.38)	0.23 (0.09–0.85)^d^	0.92 (0.38–1.18)	
**Smoking history**				
Never	14 (63.6%)	5 (62.5%)	9 (60.0%)	0.333^a^
Former	2 (9.1%)	0 (0.0%)	3 (20.0%)	
Daily	6 (27.3%)	2 (25.0%)	3 (20.0%)	
Unknown	0 (0.0%)	1 (12.5%)	0 (0.0%)	
**Additional oral care (dental floss, mouthwash)**				
Yes	16 (72.7%)	5 (62.5%)	6 (40.0%)	0.057^a^
No	6 (27.3%)	2 (25.0%)	9 (60.0%)	
Unknown	0 (0.0%)	1 (12.5%)	0 (0.0%)	
**Toothbrushing frequency per day**				
≥3 times	15 (68.2%)	6 (75.0%)	6 (40.0%)	0.046^a^
≤2 times	7 (31.8%)	1 (12.5%)	9 (60.0%)	
Unknown	0 (0.0%)	1 (12.5%)	0 (0.0%)	

^a^Fisher’s exact test, ^b^Kruskal-Wallis One Way Analysis of Variance on Ranks, ^c^Two moderate and 13 severe chronic periodontitis patients, ^d^These values were determined based on data from 7 patients due to missing data in one patient.

**Table 5:** Validation of machine learning classification with new data set (n = 45, 22 healthy controls, 8 slight, 15 moderate or severe chronic periodontitis patients).

Group		Model	Feature combination	Accuracy	Balanced accuracy	Sensitivity	Specificity
1	H vs. M-S	Neural Network	*Tf + Pg + Pi + Fn + Pa + Cr + Td*	0.84	0.85	0.93	0.77
	H vs. M-S	Random Forest	*Tf + Pg + Fn + Td+ Ec + Cr*	0.86	0.88	0.93	0.82
	H vs. M-S	Support Vector Machine	*Tf + Pg + Pi + Pa + Td*	0.78	0.81	0.93	0.68
	H vs. M-S	Regularized Logistic Regression	*Tf + Pg + Pi + Cr*	0.81	0.83	0.93	0.73
			**Average**	**0.82**	**0.84**	**0.93**	**0.75**
2	H vs. Sli-M-S	Neural Network	*Tf + Ec + Pg + Pa + Td*	0.69	0.69	0.74	0.64
	H vs. Sli-M-S	Random Forest	*Tf + Ec + Aa + Pg + Pa + Cr*	0.76	0.75	0.78	0.73
	H vs. Sli-M-S	Support Vector Machine	*Tf + Cr + Pa + Pi + Fn*	0.67	0.66	0.87	0.45
	H vs. Sli-M-S	Regularized Logistic Regression	*Tf + Cr + Pg + Pa + Aa + Fn + Pi*	0.71	0.71	0.83	0.59
			**Average**	**0.71**	**0.70**	**0.80**	**0.60**
3	H vs. Sli	Neural Network	*Tf*	0.63	0.51	0.25	0.77
	H vs. Sli	Random Forest	*Tf + Pg*	0.73	0.66	0.50	0.82
	H vs. Sli	Support Vector Machine	*Tf +Td*	0.63	0.51	0.25	0.77
	H vs. Sli	Regularized Logistic Regression	*Tf + Aa + Td*	0.60	0.53	0.38	0.68
			**Average**	**0.65**	**0.55**	**0.34**	**0.76**

### Visualization of the Best Bacterial Combinations With T-Distributed Stochastic Neighbor Embedding (t-SNE) Using a Random Forest Model

**Figure 4:**
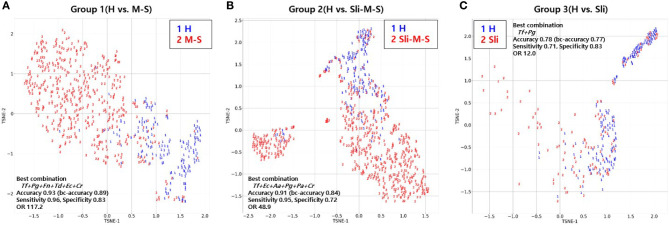
T-distributed Stochastic Neighbor Embedding (t-SNE) plot of the 692 subjects using random forest model. **(A)** t-SNE plot of periodontally healthy people (H) and patients with moderate or severe chronic periodontitis (M-S); **(B)** t-SNE plot of periodontally healthy people (H) and patients with slight, moderate or severe chronic periodontitis (Sli-M-S); **(C)** t-SNE plot of periodontally healthy people (H) and patients with slight chronic periodontitis (Sli).

To visualize the high dimensional dataset using the best combinations of bacterial copy numbers, the t-SNE plots of 692 subjects were obtained using a random forest model that showed the highest accuracy in the independent validation step shown above (**Figure 4**). Groups including moderate or severe periodontitis appear to be distinct from healthy controls (**Figures 4A, B**), while slight periodontitis appears to partially overlap with the healthy controls (**Figure 4C**).

## Discussion

In this study, we analyzed 144 healthy controls and 548 chronic periodontitis patients and demonstrated that a multivariate machine learning algorithm based on nine salivary bacterial copy numbers is able to predict the severity of chronic periodontitis.

The accuracy of our machine learning models ranged from 0.78 to 0.93 depending on the severity of the cases (**Table 3**). Balanced accuracy, which corrected for differences in population numbers between groups, were from 0.77 to 0.88. Chen et al. reported that four models classified the samples into healthy controls and periodontitis patients (moderate or severe) with 0.88–0.93 average accuracy for 76 subjects (Chen et al., 2018). We yielded similar results in the four models that classified the subjects into healthy controls and moderate or severe chronic periodontitis patients with an average accuracy of 0.93 in our large data set, even when the pathogens used were different (Group 1). We discovered that *P*. *gingivalis* and *T*. *forsythia* were included in the most statistically robust set of bacteria for each condition. These “red complex” pathogens are well known for their strong connection to periodontal breakdown (Socransky et al., 1998).

Our predictive models classified the healthy controls and all chronic periodontitis patients with 90% average accuracy (Group 2). In addition, we report, to our knowledge, the first effort to distinguish between healthy controls and slight periodontitis by machine learning algorithms based on salivary bacterial copy number (Group 3). Although the average accuracy is the lowest compared to other classification groups, Group 3 (H vs. Sli) showed an average AUC of 0.82 (average sensitivity and specificity = 0.71 and 0.84, respectively). The optimal number of bacteria for the best feature combinations was one to three, especially those including one or two red complex pathogens, and with each addition of bacterial species, the accuracy declined (**Figure 3**).

The copy number of *Aa* was not different in healthy controls and periodontitis patients, although the detection frequency was statistically significant (**Figure 2** and **Table 2**). We previously reported that both the detection rate and the copy number of *Aa* were not significant in 170 subjects (Kim et al., 2018), but the detection frequency was significant in this study. This might be due to the larger sample size and the adjustment of technical detection criteria. Nevertheless, the copy number of *Aa* was still not significant in 692 subjects. In fact, *Aa* was incorporated within some of the best feature combinations of Group 2 or 3 (**Table 3**). Although the amount of *Aa* does not differ statistically, the predictive accuracy may have increased due to a slight increase in *Aa* in periodontitis patients compared to the healthy controls.

When the subjects were divided into two classifications (healthy to slight (H-Sli) vs. moderate to severe (M-S)), the average accuracy and balanced accuracy were 0.85 and 0.81 (AUC= 0.89, OR: 33.93), respectively (data not shown). Compared to Group 3 (H vs. Sli), the average accuracy and balanced accuracy were better by 7% and 4%, respectively. In addition, we used the prediction models for multi-class classification (H vs. Sli vs. M-S) and the average accuracy and balanced accuracy were 0.79 and 0.71, respectively (data not shown). These were lower than those of Group 2 (0.90 and 0.82 in H vs. Sli-M-S). Sometimes initial clinical attachment loss and alveolar bone loss are not sufficiently recognizable in slight periodontitis, making the diagnosis and evaluation difficult for the dentist (Tonetti et al., 2018). Group 3 showed the lowest accuracy compared to other groups, which means that the diagnosis of slight periodontitis is still difficult.

Interestingly, the first high-accuracy pathogen in Group 1~3 was *Tf*, which yielded a higher odds ratio and accuracy when combined with other pathogens (**Supplementary Figures S2** and **S3**). The detection rate of *Tf* in healthy controls was less than half (49.3%) and increased according to the severity of periodontitis (P-value < 0.001 in chi-squared tests). Likewise, the copy number of *Tf* also increased dramatically as the progression of periodontitis worsened (P-value < 0.001 in Kruskal-Wallis test).

Furthermore, we performed validation for the four classification models with salivary bacterial copy numbers from 45 newly obtained samples (**Tables 4** and **5**). The overall accuracy here for all groups was lower than that of models in the training sets. In particular, slight periodontitis patients of Group 3 were separated from the healthy controls, with an average accuracy and a balanced accuracy of 0.65 and 0.55, respectively. Among the models in Group 3, the random forest model showed the highest performance (accuracy of 0.73, balanced accuracy of 0.66, sensitivity of 0.50, and specificity of 0.82). Overall, the decreased accuracy in the independent validation step may be due to the insufficient number of patients with slight periodontitis as well as the number of samples per group (22 healthy controls, 8 slight, 2 moderate, and 13 severe periodontitis).

Lastly, we visualized the best combination from the random forest model using t-SNE to see how different the severity of periodontitis for 692 subjects was (**Figure 4**) because this model showed the highest accuracy for all groups despite analyzing a small number of independent validation samples (**Table 5**). Groups including moderate or severe periodontitis were separated from the healthy controls with an accuracy of 0.93 and 0.91, respectively (**Figures 4A, B**). Although those with slight periodontitis in Group 3 appear to partially overlap with the healthy controls in contrast to other groups, the accuracy was 0.78 when the combination of two pathogens, *Tf + Pg*, were used (**Figure 4C** and **Table 3**).

Nonetheless, there are some limitations to this study. First, only nine common pathogens were selected based on a previous study (Kim et al., 2018). However, some recent studies based on oral metagenomics data have reported the use of diverse microbial species in discriminating caries or periodontitis from healthy samples (Alcaraz et al., 2012; Wang et al., 2013). Second, host-derived salivary biomarkers were not evaluated. Salivary proinflammatory cytokines and enzymes play crucial roles against oral microorganisms and could serve as biomarkers (Kinney et al., 2011; Wu et al., 2018). Third, our study is based on a limited number of samples collected from a single center, creating the possibility of bias, so validation using orthogonal datasets is required. In addition, the potential prognostic value for disease progression and the response to treatment in periodontal patients should be validated in longitudinal studies. Despite these limitations, our results still provide an important basis for further studies.

In conclusion, we applied four machine learning algorithms to compare healthy controls and patients with differing severities of periodontitis, based on the abundance of salivary bacterial copy number. Our findings suggest that the optimal combination of salivary bacteria could be a biomarker with the ability not only to differentiate between healthy controls and periodontitis patients but also classify the severity of the periodontitis cases. Large and well-designed studies are the key to identifying the novel pathogen markers from oral microbiota associated with the various degrees of severity of periodontitis and also to validating the performance of machine learning models. These studies may provide a better understanding of the pathogenesis of periodontitis while improving the accuracy of diagnoses of periodontitis cases with varying degrees of severity.

## Data Availability Statement

The original contributions presented in the study are included in the article/**Supplementary Materials**. The qPCR data generated or analyzed in the present study are included in the supplemental material (**Table S1** and **S2**). Further inquiries can be directed to the corresponding authors.

## Ethics Statement

The studies involving human participants were reviewed and approved by Institutional Review Board, Pusan National University Dental Hospital. The patients/participants provided their written informed consent to participate in this study.

## Author Contributions

E-HK: Conceptualization, data curation, formal analysis, funding acquisition, investigation, methodology, project administration, resources, visualization, and writing. SK: Conceptualization, formal analysis, investigation, methodology, programming, validation, visualization, and writing. H-JK: data curation, formal analysis, investigation, methodology, and resources. H-OJ: Conceptualization, methodology, programming, validation, and visualization. JL: Formal analysis and visualization. JJ: Methodology and programming. J-YJ and YS: Data curation. JK: Funding acquisition, project administration, and resources. AP: Methodology, validation. J-YL and SL: Funding acquisition, resources, and supervision. All authors contributed to the article and approved the submitted version.

## Funding

This work was partly supported by the Technological Innovation R&D Program (C0445482), funded by the Small and Medium Business Administration (SMBA, South Korea). This work was also partly supported by the Next-Generation Information Computing Development Program of the National Research Foundation of Korea funded by the Ministry of Science and ICT (NRF-2016M3C4A7952635). This work was also partly supported by the National Research Foundation of Korea (NRF) grant NRF-2017M3A9B6062026, funded by the Korean government.

## Conflict of Interest

SK and SL are co-inventors on a patent application related to this research (no. 10-2019-0095319, 06 August 2019). E-HK, YS, and JK were employed by the company Helixco Inc.

The remaining authors declare that the research was conducted in the absence of any commercial or financial relationships that could be construed as a potential conflict of interest.

## Supplementary Material

Supplementary Figure 1 Receiver operating characteristic (ROC) curves of the four predictive models based on the best bacterial combinations.

Supplementary Figure 2 Accuracy and balanced accuracy of single feature (*Tf*) versus feature combination.

Supplementary Figure 3 Odds ratio with 95% CI of single feature (*Tf*) versus feature combination in Group 1 to Group 3.
