# Supplementary figures and images for: Prediction of Chronic Periodontitis Severity Using Machine Learning Models Based On Salivary Bacterial Copy Number
Source: Front Cell Infect Microbiol. 2020 Nov 16;10:571515. doi: 10.3389/fcimb.2020.571515 (PMC7701273; doi:10.3389/fcimb.2020.571515)

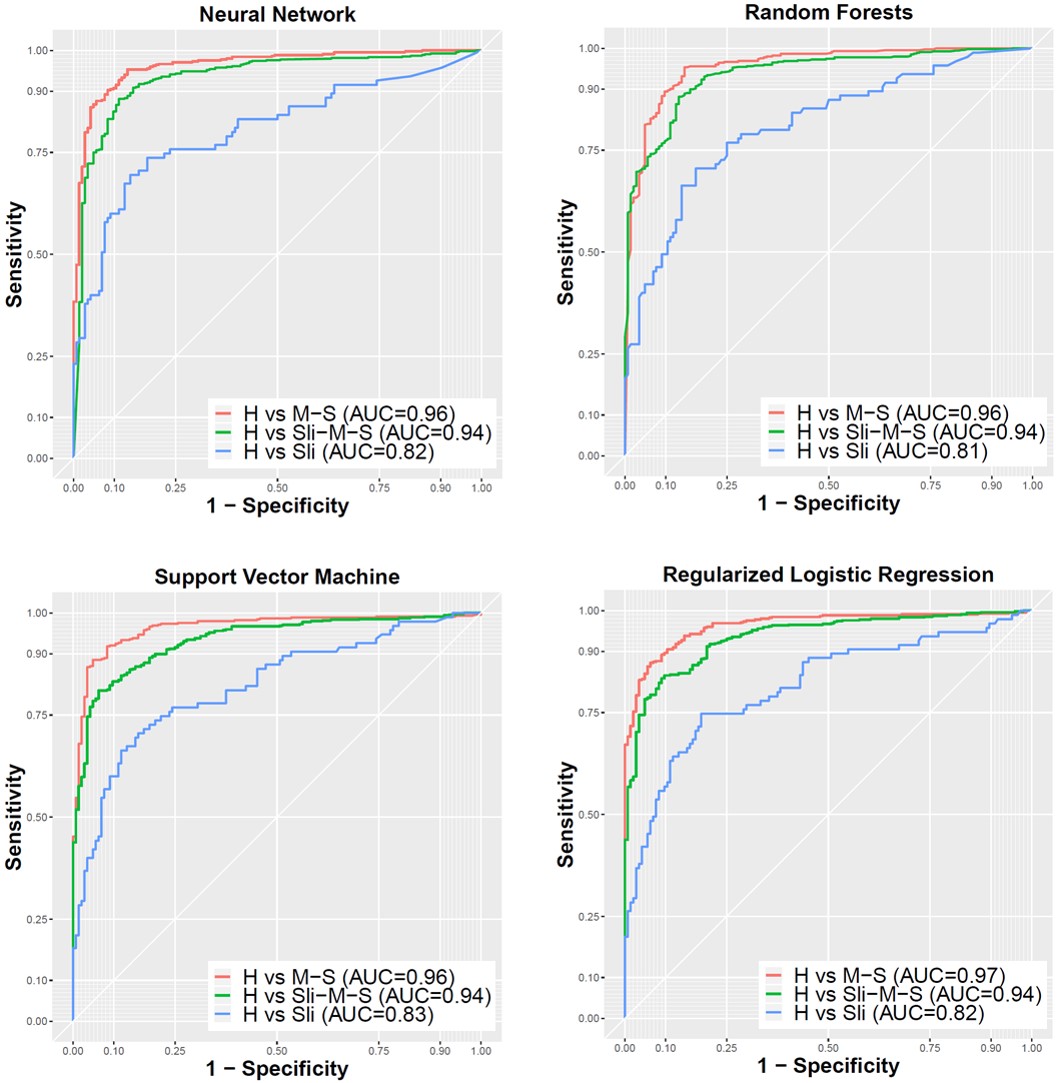

Supplement: Supplementary Figure 1 — Receiver operating characteristic (ROC) curves of the four predictive models based on the best bacterial combinations. [file Image_1.jpeg]

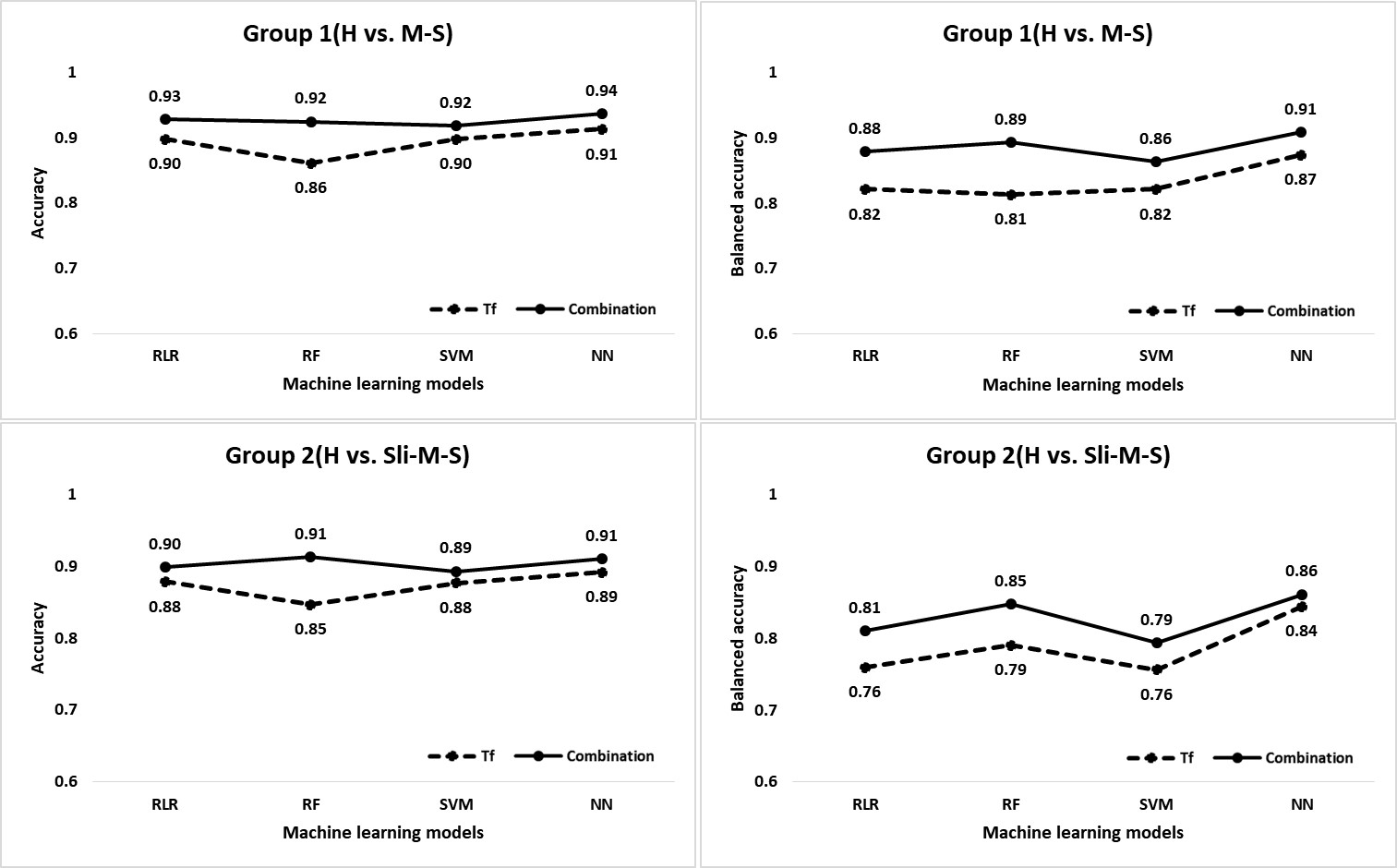

Supplement: Supplementary Figure 2 — Accuracy and balanced accuracy of single feature (Tf) versus feature combination. [file Image_2.jpeg]

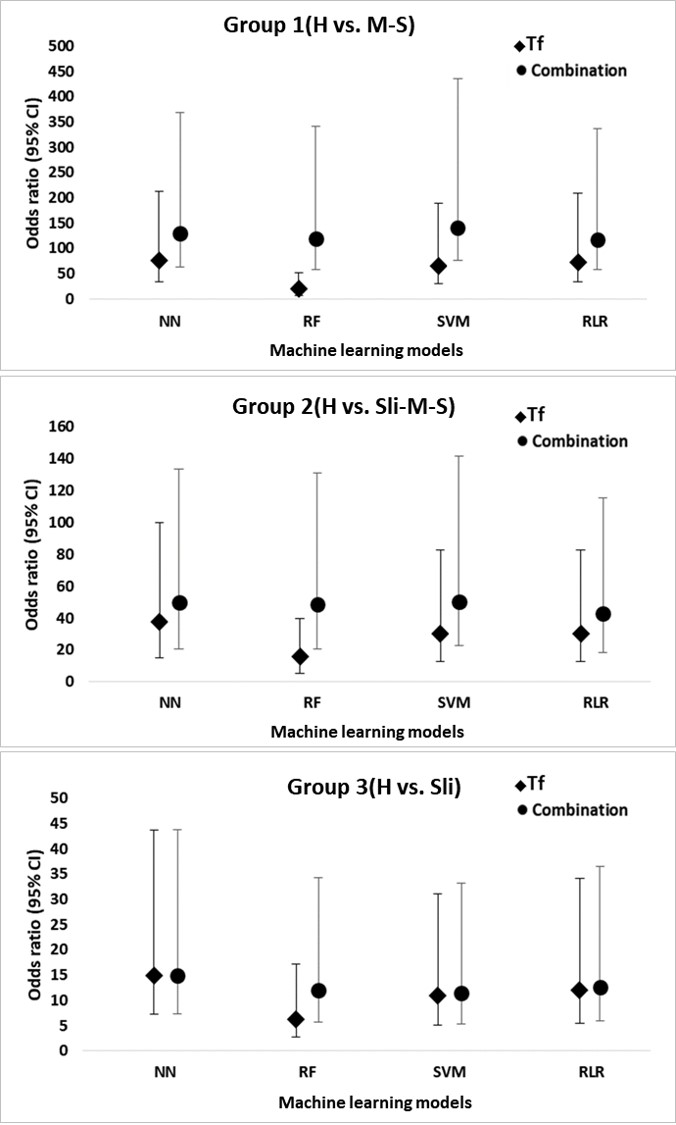

Supplement: Supplementary Figure 3 — Odds ratio with 95% CI of single feature (Tf) versus feature combination in Group 1 to Group 3. [file Image_3.jpeg]
